# Assessing the quality of annotations in asthma gene expression experiments

**DOI:** 10.1186/1471-2105-11-S9-S8

**Published:** 2010-10-28

**Authors:** Ronilda Lacson, Michael Mbagwu, Hisham Yousif, Lucila Ohno-Machado

**Affiliations:** 1 Decision Systems Group, Brigham & Women’s Hospital, Harvard Medical School, Boston, MA, USA; 2 Ohio State University, Columbus, OH, USA; 3 University of Arizona, Tucson, AZ, USA; 4 Division of Biomedical Informatics, University of California San Diego, La Jolla, CA, USA

## Abstract

**Background:**

The amount of data deposited in the Gene Expression Omnibus (GEO) has expanded significantly. It is important to ensure that these data are properly annotated with clinical data and descriptions of experimental conditions so that they can be useful for future analysis. This study assesses the adequacy of documented asthma markers in GEO. Three objective measures (coverage, consistency and association) were used for evaluation of annotations contained in 17 asthma studies.

**Results:**

There were 918 asthma samples with 20,640 annotated markers. Of these markers, only 10,419 had documented values (50% coverage). In one study carefully examined for consistency, there were discrepancies in drug name usage, with brand name and generic name used in different sections to refer to the same drug. Annotated markers showed adequate association with other relevant variables (i.e. the use of medication only when its corresponding disease state was present).

**Conclusions:**

There is inadequate variable coverage within GEO and usage of terms lacks consistency. Association between relevant variables, however, was adequate.

## Background

The Gene Expression Omnibus (GEO) project was initiated by the National Center for Biotechnology Information (NCBI) to serve as a repository for gene expression data [1,2]. In addition to GEO, there are several other large-scale genetic databases, including ArrayExpress, the Center for Information Biology Gene Expression Database (CIBEX), and the Stanford Tissue Microarray Database (TMAD), each with similar structures and purposes [3-5]. Currently, GEO contains over 17,000 experiments and 400,000 samples. There has been an ever growing interest in large microarray repositories for several reasons: (a) Microarray data are required by funding agencies and scientific journals to be made publicly accessible; (b) such repositories enable researchers to view data from other research groups; and (c) with proper pre-processing, such repositories may allow researchers to formulate and test hypotheses in a relatively inexpensive manner [6]. There are also other advantages in pooling data from several studies, such as providing quantitative researchers with access to a diverse dataset to verify their algorithms, and to check consistency of results over a large dataset [7,8].

Although GEO constitutes a major advance to promote data sharing, it is not without its imperfections, particularly related with the annotation of data. In late 2001, the Minimum Information About a Microarray Experiment (MIAME) standard was developed by the Microarray Gene Expression Databases Society (MGED) in an effort to standardize the way data were entered in GEO and other public repositories [9]. Members of the consortium realized that gene expression data were only useful if it could be put “…in the context of a detailed description of conditions under which they were generated” [9]. This led to MIAME’s basic tenets that all data be recorded with enough information and detail to allow samples to be compared to others (and therefore could be verified for reproducibility), as well as making information accessible for data mining and other automated means of analysis. Specifically, MIAME made provisions for the use of controlled vocabulary (as opposed to free-text fields), as well as descriptions of experimental design, array design, samples, hybridizations, measurements, and normalization [9]. MIAME does not specify the use of any particular terminology. However, the use of standard controlled vocabularies is desirable to promote data exchange.

Several authors, however, have noted flaws in the practical use of the MIAME standard by researchers. Galbraith points out that the MIAME standard is lacking in informational content, to the point that a researcher will have difficulty understanding relevant factors that contributed to the results generated from the data [10]. Shields brought out another important point, namely, that although MIAME may be a good standard for reporting data, it still falls short of ensuring that various laboratories have uniform experimentation techniques [11].

Even after MIAME was implemented, Ioannidis et al. showed that while some microarray experiments followed the MIAME standard, many others did not, and even those that did often had insufficient information to recreate experiments and reproduce results [12]. In addition, because the use of controlled terminology is not a requirement for MIAME compliance, sample annotations and experimental design descriptions are deposited as free text. Thus, it is difficult to ensure and enforce compliance with MIAME standards.

The goal our study was to assess the adequacy of documented information describing data samples in GEO, specifically in the asthma domain.

## Method

We explored the quality of data deposited in GEO for 17 asthma studies. We utilized a toolkit developed for analysis of gene expression data in GEO, DSGeo [13]. DSGeo contains a browser that renders the studies in GEO available for text queries. This browser returns research studies when samples or platforms contain the search term within sample data or experimental design descriptions. In order to identify all appropriate studies for which there were samples in GEO, a text search for the term “asthma” was used and all studies that were retrieved were annotated and analyzed. For each study, all samples were annotated using domain-specific pre-defined variables. Methods for identifying variables and re-organizing the information from GEO in a relational database were previously described [14,15].

**Figure 1:**
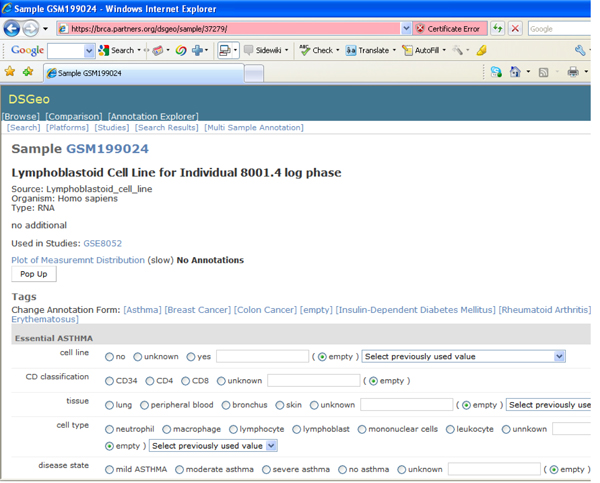
Asthma annotation form

The annotation tool used for this research was developed to facilitate human annotation by allowing easy access between the data descriptions and measurements that were downloaded from GEO and appropriate scientific publications from Pubmed [13]. The annotators are able to read the study descriptions that researchers deposited in GEO, as well as individual sample descriptions, attached tables and supplementary documents. Each annotator is given access to full-text electronic copies of articles, whenever available, to aid in annotation. Figure 1 illustrates the annotation tool used for assigning values to specified variables for every sample. Cell line would have values “yes”, “no” or “unknown”.

### Annotation training

There were two annotators for this study, both trained biology students. They underwent an orientation period to familiarize themselves with the GEO database as well as tools that were developed to examine the data samples. They were given time to learn how to use the web interface, specifically the free-text dialog boxes as well as the standard radio buttons and drop-down menus used for annotating most of the variables. In addition, they were acquainted with the organization of the database, in a way that they would be able to access any supplemental information essential to completing an annotation. Techniques such as batch sample annotation and annotation grouping were also introduced in order to maximize the efficiency of annotation. To practice, the annotators (undergraduate students majoring in biology) were then assigned to annotate a domain which had already been previously annotated, the breast cancer domain [14].

As in previous experiments, written instructions were given to provide the students with an extensive list of definitions for variables specific to breast cancer and guidelines on how to annotate them. Once a student completed the previously annotated breast cancer domain, the results were analyzed based on the quality of annotation as well as timeliness of completion. Upon satisfactory completion, each student was considered eligible to begin data annotation for asthma samples. Using the same sequence of training steps used for annotating samples in the breast cancer domain, students were assigned asthma studies and samples for annotation.

### Inter-annotator agreement

Inter-annotator agreement was measured using strict agreement, which measured the total number of annotations that were exactly alike between the two annotators. Kappa statistical measure was also used to measure the inter-annotator agreement, taking into account the probability of agreement due to chance [16]. In the case of breast cancer, the quality of annotation was measured by comparing the new annotations to values previously established [14].

Seventeen asthma studies were annotated. The studies consisted of 918 samples, ranging from 404 samples in the largest study to 2 samples in the smallest one. All of the studies were annotated using 26 different variables. Inter-annotator agreement was measured between the two student annotators.

### Adequacy of documentation

We examined three primary measures (coverage, consistency, and association) to evaluate the quality of textual data accompanying data samples. Coverage was defined as the number of variables that have known values relative to a sample set. For example, if only 10 of 100 total samples had known values for the variable ‘gender’, then coverage was 10%. Consistency was defined as the lack of variation in term usage for data reporting and publication (e.g., consistently using the generic name of medications in any data provided by the researchers, rather than using the trade name in one and the generic name in another). Association was assessed by measuring how often appropriate medication use and asthma diagnosis co-occurred. It was expected that asthma medications should be used only for samples/patients that had the diagnosis of asthma.

## Results

### Breast cancer (training data)

A total of 604 breast cancer samples from two studies were re-annotated by the two students during the training phase. There were 41 markers for breast cancer, as described in reference [14]. The results of these annotations are shown in Table 1.

**Table 1:** Inter-annotator agreement for the breast cancer domain performed by students in comparison to previous annotation

Annotator	Agreement Type	% Agreement	Kappa
Annotator 1 N=318	Strict	79.5	0.524
Annotator 2 N=286	Strict	80.9	0.401

The 318-sample study contained 12,739 annotations in total. The total amount of annotations is less than what would have been 318 samples multiplied by 41 variables because of non-mandatory fields. For instance, cancer staging is not a mandatory field for one of two (or more) staging methods used in practice (e.g. TNM, Duke classification). Thus, leaving one of these fields blank would lead to fewer annotations for a given sample. Of the 12,739 annotations, the first annotator strictly agreed with the previous annotator on 10,127 annotations, or 79.5% of the time. Kappa showed moderate agreement, which was assessed to be sufficient. The 286-sample study had a total of 10,589 annotations. The second annotator agreed with the previous annotator in 8,567 annotations, or 80.9% of the time. The kappa measure was 0.401, also showing moderate agreement. This training was assessed to be sufficient for the students to annotate a new domain.

### Asthma

The asthma domain contained 20,640 annotations. Strict inter-annotator agreement was measured between the two student annotators, as well as kappa inter-annotator agreement. The results of this study are noted in Table 2, which shows excellent inter-annotator agreement between the student annotators. There was 93% strict agreement between annotators. The kappa measure was 0.859, which corresponds to almost perfect agreement.

**Table 2:** Inter-annotator agreement for the asthma disease domain

Agreement Type	% Agreement	Kappa
Strict	93	0.859

There was an early attempt by the NCBI to have annotations of the data within GEO with the creation of GEO Data Sets (GDS).(1) This effort was limited, and there remains a majority of the GEO database that is not annotated. Out of the 17 asthma studies examined, only 4 had GDS annotations. These four studies contained a total of 212 samples. The studies had an average of 2.333 annotations per sample. The variables that were annotated in GDS included “agent”, “disease state”, “time”, and “other”. The coverage (or the percent of the samples containing a value for a given variable) was examined, and the results are displayed in Table 3. Table 4 shows all the studies’ goals and the number of samples in each of the 17 annotated studies.

**Table 3:** Coverage of Asthma variables in GDS

	GSE 470	GSE 473	GSE 3183	GSE 3004	Total
Agent	100%	0%	100%	100%	17.4%
Disease State	100%	100%	0%	0%	88.2%
Time	100%	0%	100%	0%	12.7%
Other	0%	100%	0%	0%	82.5%
No. of Samples	12	175	15	10	212

**Table 4:** Annotated GEO asthma studies

Study	No. of Samples	Topic/Title
GSE8052	404	Determinants of susceptibility to childhood asthma
GSE473	175	Defining diagnostic genes from purified CD4+ blood cells that have specific diagnostic profiles
GSE4302	118	Profiling of airway epithelial cells
GSE3184	40	Murine airway hyperresponsiveness
GSE483	39	Allergic response to ragweed
GSE1301	24	Mechanisms by which IL-13 elicits the symptoms of asthma
GSE8668	24	Effects of exercise on gene expression
GSE6858	16	Expression data from experimental murine asthma
GSE3183	15	Early cytokine-mediated mechanisms that lead to asthma
GSE470	12	Asthma exacerbatory factors
GSE9465	12	Pulmonary responses to ambient particulate matter
GSE3004	10	Effects of allergen challenge on airway cell gene expression
GSE2276	9	Effect of PGE receptor subtype agonist on an asthma model
GSE476	8	Ozone effect on airways hyperpermability
GSE481	5	Allergen-induced goblet cells
GSE477	5	Alternatively activated macrophages
GSE2955	2	Transcriptional activation of AhR pathway in keratinocytes

There is relatively inconsistent coverage of the GDS data within GEO. There was a wide gap between the variable with the highest (88.2%) and the lowest (12.7%) percent coverage. Both “Agent” and “Time” variables were the least covered, while “Disease State” had good coverage.

From GEO, a total of 918 samples were examined. 26 variables in each sample were annotated, which was a significant increase from the 4 variables that GDS currently covers. There were a total of 20,640 annotations, but of these variables, only 10,419 had known values, with a variable coverage of 50.5% for all variables that were annotated.

As shown in Table 5, the majority of variables were found to be poorly covered, with the variable (“Tissue”) having a coverage of 56.1% and the poorest covered variable (“Race”) with 0.2%. These results were typical of most other variables, and illustrate the difficulties in reusing data that are currently deposited into GEO.

**Table 5:** Selected examples of coverage in annotated variables

Variable	# Unknown	Coverage (%)
Race	916	0.2
Age	855	6.9
Family history	729	20.6
Systemic steroid	729	20.6
Genetic deficiency	729	20.6
Steroid inhaler	697	24.1
Disease frequency	627	31.7
Gender	489	46.7
Atopic	425	53.7
Tissue	403	56.1
Challenge	0	1.0

The consistency of the studies in the asthma domain was also measured. In one such study (GSE4302), the data for 32 asthmatics randomized to a placebo-controlled trial of fluticasone propionate were examined. The authors use the generic name “fluticasone propionate” within both the abstract and the manuscript; however the trade name “Flovent” is used within the data deposited within GEO. Inconsistencies such as this could prove problematic when trying to repeat a medication trial experiment, since, for example, there can be subtle differences in medications that differ in trade name but represent the same chemical entity.

Within the studies examined, an association between asthma diagnosis status and the use of various medications (or the lack thereof) was assessed.

Table 6 shows the different variables of asthma states as well as the medication variables. Using Fisher’s exact test, a strong association between asthma diagnosis and the annotation of a medication variable (either “short acting beta agonist”, “steroid inhaler”, or “systemic steroid”) was established, as shown in Table 7. This result showed that while variable coverage may be scarce, there was appropriate use of medication variables only when a corresponding asthma diagnosis was present

**Table 6:** Association of medication use and asthma severity variables

	No steroid inhaler	No beta agonist	Short acting beta agonist	Steroid inhaler	Systemic steroid	Others
Mild asthma (105)	0	0	10	0	0	0
Moderate asthma (39)	0	0	0	19	0	0
Severe asthma (80)	0	0	0	0	0	0
No asthma (255)	138	138	0	0	0	0

**Table 7:** Association between asthma diagnosis and medication use (Fisher’s Exact, p=0.000002)

	Medication Use		
**Asthma diagnosis**		Yes	No/Unknown

	Yes	29	195	
	No	0	138	

## Discussion

Gene expression repositories currently hold a large amount of data, and are continually expanding at a rapid rate [1]. Because of this, it is important to ensure that data placed into repositories such as GEO contain enough information so that they can be useful for future analysis [17,18].

We show that for four studies with GDS annotations, there were only 2.333 annotations per sample. Moreover, coverage appeared to be limited for two of the variables. This can be partly explained by annotations in GDS, which are discretionary and vary between studies even within the same domain. For example, the coverage for the variable “Agent” is low even with 100% coverage in three studies with 12, 15 and 10 samples each. The coverage is offset by 0% coverage in one big study with 175 samples. Thus, the overall coverage is diminished. It would be desirable to have more GDS annotations for more studies and samples to determine a more robust estimate of variable coverage using GDS.

For the 17 studies that we annotated, we show that there was only 50.5% variable coverage for asthma studies within GEO. The coverage is inconsistent and fluctuates between different variables. Certain variables that one might consider important for genetic asthma studies were only annotated some of the time (such as family history, with 20.6% coverage). Some of the coverage issues may have been attributed to inappropriate variables used in analysis (i.e. the use of “Race” when evaluating a murine model experiment). Future work may look at identifying variables specific to individual studies (and in a broader sense, variables specific to domains of work), and only comparing studies within those specific subsets.

This study demonstrated that there are key discrepancies in the data deposited within GEO, but also offers evidence that it is possible to re-annotate the data with relatively few resources in a short amount of time [12]. Re-annotation of sample data by two trained annotators resulted in 93% inter-annotator agreement for asthma, and 80% inter-annotator agreement for the training domain, breast cancer. Some of this improvement can be attributed to familiarity with the annotation process, resulting in committing fewer errors in annotation. In addition, there were only 26 asthma variables, compared to 41 breast cancer variables for annotation. Manually searching for values for fewer variables is an easier task, and therefore less prone to errors and discrepancies. Overall, inter-annotator agreement was excellent, providing reliable annotations to determine annotation adequacy.

Only a limited number of samples were used for evaluation of consistency (since only one study had appropriate parameters). The use of “Flovent” rather than “flucticasone propionate” could cause confusion to annotators not familiar with the field, and, more importantly, is likely to pose difficulties to data mining software that may be programmed to recognize some trade names but not others. Lack of consistency may hamper attempts to accurately extract and integrate data from GEO, but more studies are needed to better evaluate the magnitude of this problem for the whole collection. Other variables that could be evaluated for consistency would be cancer staging (if there are more than one staging systems) or presence of metastases, which should correspond to a stage four cancer. Any annotation inconsistency should be addressed with the study investigators to avoid errors in data analysis.

There was strong association of the variables defined as types of medication and the asthma disease state variable. This finding confirms that annotations of variables are appropriately associated with other relevant variables (i.e. the use of medication should only being attributed to subjects who actually have asthma, not those without). Although the sample size was small, this association was highly significant (p=0.000002), warranting further study of variables and associations.

## Conclusions

Adequate sample annotation within GEO is important for data to be usable by the scientific community. In particular, it is important for variables to be consistent and to be comprehensive and include key features of the experiment. There was inconsistency in medication name usage, which would benefit from further studies on consistency of sample annotations. We show that the coverage within GEO is inadequate in the domain of asthma, while association appears to be satisfactory.

## Authors' contributions

All authors were involved in designing the study. RL and LOM were involved in system design and development of the user interface. After the initial pilot phase, RL and LOM were involved in further variable identification and selection. MM and HY were involved in performing annotations. RL was involved in supervising the entire annotation process and evaluating annotation quality. All authors contributed to preparation of this manuscript and read and approved the final version.

## Competing interests

The authors declare that they have no competing interests.
